# Multiple slip effects on time dependent axisymmetric flow of magnetized Carreau nanofluid and motile microorganisms

**DOI:** 10.1038/s41598-022-18344-z

**Published:** 2022-08-22

**Authors:** Muazzam Faiz, Danial Habib, Imran Siddique, Jan Awrejcewicz, Witold Pawłowski, Sohaib Abdal, Nadeem Salamat

**Affiliations:** 1 Department of Mathematics, Minhaj University, Lahore, Pakistan; 2 Department of Mathematics, Khwaja Fareed University of Engineering and Information Technology, Rahim Yar Khan, Pakistan; 3 Department of Mathematics, Government College University Faisalabad, Layyah Campus, Layyah, Pakistan; 4 Department of Mathematics, University of Management and Technology, Lahore, 54770 Pakistan; 5 Department of Automation, Biomechanics and Mechatronics, Lodz University of Technology, 1/15 Stefanowskiego St., 90-924 Lodz, Poland; 6 Institute of Machine Tools and Production Engineering, Lodz University of Technology, Lodz, Poland; 7 School of Mathematics, Northwest University, No. 229 North Taibai Avenue, Xi’an, 710049 China

**Keywords:** Engineering, Nanoscience and technology

## Abstract

This presented work investigate the bio-convections effects of the magnetized time dependent axisymmetric flow of Carreau-nanomaterial performances with multiple slip effects over a stretching sheet. The momentum, heat, concentration and density of motile micro-organism are renovated into the system of equation via using well known similarity revolution. Well known Mathematical computational techniques and software (i.e. bvp4c and MATLAB) are used to draw graphical and tabular results. Velocity profile equation $f^{\prime}(\zeta )$, energy equation $\theta (\zeta )$, volumetric nanoparticles $\phi \,(\zeta )$, density motile microorganism $\chi (\zeta )$.The Carreau viscosity model is use to reduce the viscosity of fluid when $W_{e} = 0\,\,\,$ and $\,n = 0$. Besides we moderate this into power law index with $\,n = 0.5$ and $\,n = 1.5$ partial slip condition of velocity is also instigated at the surface. Gravity dependent gyrotactic nanoparticles are utilized for well observing axisymmetric flow with convective boundary layer condition and comparatively better heat transfer rate result and applicable to maximum realistic approach.

## Introduction

On the behalf of the current investigation researchers are able to say that the main objective of these Articles is to determine the multiple slip effect on time dependent MHD flow of axisymmetric with Carreau-nanomaterial performances and motile microorganisms is boost the performs in all nanofluid field with motile microorganisms with malty slip effects and axisymmetric flow. The presentation of electronics accessories is updated in these days by the progress in the minimizing of their appliances of density power associated with electronics accessories and it enhances drastically the positive effects of heat flux to improve the efficiency of electronic accessories. The degeneracy of the heat flux profile with in the electronics accessories is the updating of study in fluid thermodynamics like over heat is the causes to devalue the electronics accessories improvement level. Accordingly, it creates the hi-tech challenges for the experts and transformers of industrial fields to develop and modern effects of cooling systems to modify the best. Such as the system under the certain defined values is evacuate the heat compeers for the conservation of temperature in electronic accessories. Modern cooling systems in these accessories are mandatory to face the challenges. In these modern systems it is necessary to boost the competence and activation of thermal schemes due to the addition of nanoparticles is the vile on fluid thermodynamics. The study of the nanoparticles in fluid is take very important place, now a day the study of the nanoparticles known as nanofluid. Nanofluids are making useful the old-style thermal systems vile activation of nanoparticles. For improvement in activation energy of thermal conductivity the experts are investigate the nanoparticles.

Now a day accumulative energy need has been the result in gigantic quest for regenerate the energy cradles at the huge flat for global world. In these days the use of solar energy is the most common source of the electric energy supplies for all global domains. The source of solar energy with use of nanoparticles was introduced and advent by Hunt^1^ in 1970s. His investigation was starts with heat transfer applications and thermal conductivity to collection of solar energy with nanoparticles which is the most appropriate desire of fluid field. Scholars finished the heat transfer and the solar energy pool manners can be enhanced with the addition of nanoparticles in the base fluids. The work in viscosity and thermal conductivity is most probable upgraded by scattering the Nano encapsulation metallic units in the fluids works by Masuda et al.^2^. Choi and Eastman^3^ 1995s advised to use the nanoparticles for improve the transfer rate in heat flux. Nanofluids can be boosted with the effects of thermal conductivity is used in different engineering and applications of MHD effect to variety of nanoparticles combining with base fluids to execute the entropy process. The motility effect also kept adequate existence in heat transformation and gyrotactic phenomenon. The discussed research proposed by^4–6^. Naz et al.^7^ proposed entropy support to Walter’s- B nanofluid to investigate the motility of bio molecules inside the nanofluid. Also, many researchers have been made their efforts on the nanofluid with Buongiorno and Tiwari Das model^8–15^.

The current consideration for increasing of energy sources and energy efficiency for industrial work we really use non-Newtonian viscoelastic Carreau Nanofluid flow by all means like the extrusion of polymer sheet for draw of dyeing of polymer films. In other words, we considered the volume of current graft has been completed in order of non-Newtonian fluids field and such as further is wanted in the range of non-Newtonian fluid mockups. There are in number of researchers come forward to search the lot of extensions about the new models in power-law index under non-Newtonian fluid. These boundaries of the power-law index, exclusively for lowest and very huge amount of shear levels, are considered in additional viscosity ideal known as Carreau-nano fluids ideal model. The Generalized kind of Newtonian fluid is Carreau fluid which depends on the viscosity shear level. To describe the action of huge shear level of viscosity rate we use the study of Carreau fluid and the 1^st^ time introduced this rheological equation in molecular links theory by Carreau^16^. Naz et al.^17^ scrutinize rotating disk to investigate the Eyring-Powel effect on bio molecules and explained thermal thermal transformation. Numerical analysis of sisko fluids to explore entropy generation and obtaining high proficiency heat transformation is elaborated by Naz and her co-worker^18^. Naz^19^ numerated the basic terms to elaborate thermal factors upon motile microorganism over viscoelastic nanofluid. Ayub et al.^20^ disclosed the aspects of all the basic parameters required for the propagation of heat transfer over magnetized Carreau nanofluid. Energy exchange featuring infinite shear rate viscosity model of Carreau nanofluids to examine radiation and heat transformation towards the poles is discussed by Shah^21^. Ayub et al.^22^ analyzed the configured approach to cross-nanofluids to examine spectral relaxation and velocity slip effect by applying magnetized inclination effect. Shah et al.^23^ presented the impact of magnetic dipole around cross nanofluids to experience the effect of heat transportation over cylindrical panels. Ayub et al.^23^ displayed an analysis on heat dissipation with Lorentz forces over cross nanofluids taking time as parameter. Higher order chemical process for heat dissipation over magnetized cross fluids have been presented by Shah et al.^24^. Three dimensional impact of same fluid over stretching sheet is applied to examine the velocity slip effect Wahab et al.^25^. The heated surface flow on MHD cross flow with nonuniform heat sink/source is disclosed by Ayub et al.^26^.

Impact of boundary layer flow and heat transfer for Carreau nanofluid is being discussed for stretching sheet by Khan^27^. Axisymmetric flow of Carreau nanofluids is disclosed by Khan^28^. The numerical exploration of heat transfer rate and flow of nanofluid is discussed by Khan and Alshomrani^29^. Unsteady stagnation-point flow and heat transfer is calculated over a permeable sheet by Basir et al.^30^ disclosed the study of heat sink/source and heat dissipation over three dimensional flow of Carreau nanofluid. Phenomena about bio-convection is arises when the tiny living orgasms which are heavier than water can be found in the form of swimming particles upward normally are known as microorganisms. As we know that the algae are the gyro tactic microorganisms which can swim to upward direction lead to focus in superior position on the layer of fluid which causes a huge density hierarchical that displayed unbalanced. The discussion about nanofluid bio-convection is styled the various design creation and density stratification convinced by the coincident interface of the debated self-propelled buoyancy forces about denser microorganisms. Modified Bourgrinoes model is applied to investigate heat distribution and flow of carreau nanofluid about wedge shaped geometry^31,32^.

The multiple slip with Stefan blowing fluid on buoyancy driven of nanofluid in the presence of bio-convection effects over a moving plate was investigated by Uddin et al.^33^. The work about bio-convection over vertical plate with special effects of convective boundary layer in MHD is developed by Uddin et al.^34^. Khan et al.^35^ works on gyrotactic microorganisms in boundary layer flow of nanofluid with vertical plate and magnetic field. Kuznetsov^36^ used oxytactic microorganism with convective boundary conditions on thermos-bio-convection porous layer. The numerical investigation of nanofluids are explore with different body forces presented by^37,38^. In MHD parabolic flow of Williamson and Casson fluids in the presence of nanofluid, Ali et al.^39–41^ presented the numerical explanations of non-Newtonian nanofluids with gyrotactic microorganisms via finite element method.

The effect of multiple slips on time dependent MHD flow of axisymmetric with Carreau-nanomaterial performances and motile microorganisms is explored in this study. Nanoparticles usually settle down while in the base fluids and the cause dies. So in this situation we have bio molecules, basically these are living organisms from the specie of algae, fungi and other type of bacteria that keep on moving, these create conductivity effect and heat conductive alive again. This is how heat transfer rate can be expected to be rise again. This phenomenon helps in achieving the goal. Moreover, this consideration over convective boundary conditions makes the under-discussion problem more realistic physically and numerically in all value able appearance. Appropriate changes in this model are used to alter the valuable nonlinear PDE’s to nonlinear ODE’s. The numerical values of the given model are verified for both cases of power law index $n = 0.5\;{\text{to}}\;1.5$ and show a decent association with previous model of fluids.

## Developed model

**Figure 1:**
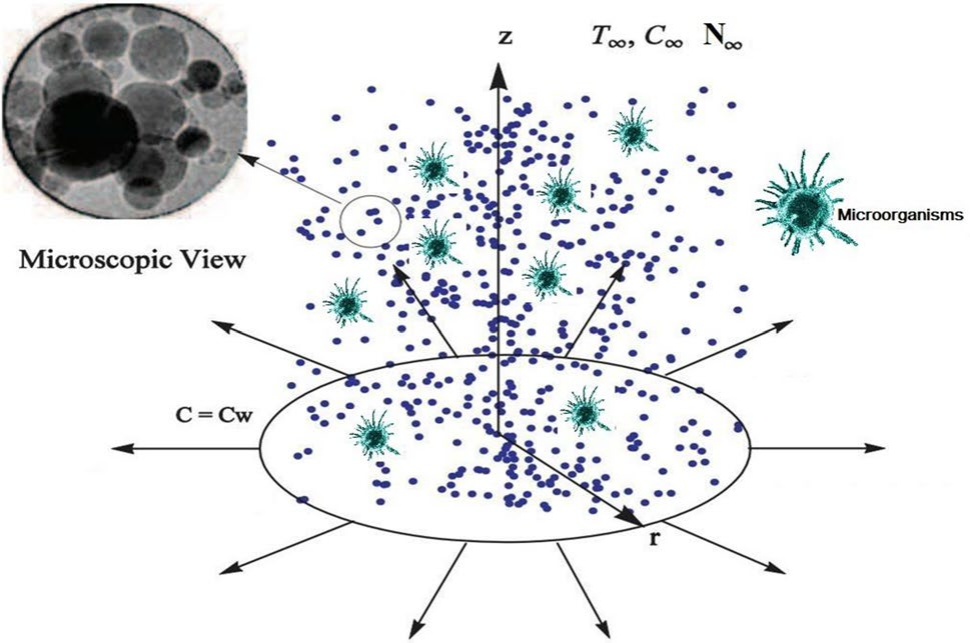
Flow configuration with motile microorganisms.

In this present unruly of multiple slip effect on time dependent MHD flow of axisymmetric with Carreau-nanomaterial performances and motile microorganisms is considered. The stretching velocity is directly comparative to the radius ${\dot{r}}$ at the origin (0,0) with $\wedge_{1} \,,\, \wedge_{2} > 0$ non-moving parameters taking the dimensions about time $\left[ {T^{ - 1} } \right]$ just because the stretching condition of sheet also with stretching velocity $U_{w} ({\dot{r}},\dot{t},\dot{z}) = {{ \wedge_{1} {\dot{r}}} \mathord{\left/ {\vphantom {{ \wedge_{1} {\dot{r}}} {1 - \wedge_{2} \dot{t}}}} \right. \kern-\nulldelimiterspace} {1 - \wedge_{2} \dot{t}}}$ in the directional of radial side. In the upper half plane flow seems of fluid and the sheet is corresponding with the plane $\dot{z} = 0$. For measured depiction, we consider the three-dimensional polar coordinate system in cylinder $({\dot{r}},\theta ,\dot{z})$. A derivational sloping magnetic strength in field $B(t) = \,{{D_{0} } \mathord{\left/ {\vphantom {{D_{0} } {\sqrt {1 - \beta t} }}} \right. \kern-\nulldelimiterspace} {\sqrt {1 - \beta t} }}$ is carried out in the direction of $\dot{z}$ side, $\forall$ $D_{0} > 0$ is a constant of magnet (see Fig. 1). The induced magnetic field is neglected because we take a very small value of magnetic Reynolds number. Newly devised models $N_{b} ,\,N_{t}$ are integrating the effects of nanofluid ‘Brownian motion and thermophoresis’ is utilized respectively. Moreover, $\lambda$ is expose the partial slip condition of velocity at the outward is also carried out and for demand to change in temperature a frenzied fluid is used, with temperature $T_{w} (x)$ and the transfer mode of convective heat flux that’s delivered $h_{f}$ as transfer of heat coefficient also in sheet. Furthermore, constant concentration $C_{w} (x)$ with $C_{w} (x) - C_{\infty }$ is also use at the surface of the sheet. Additionally, the surface of sheet is at constant motile microorganism $N_{w} (x)$ with $N_{w} (x) - N_{\infty }$. For the unsteady 2-D axisymmetric flow, the velocity $f$, boundary layer thickness $\theta$ nanoparticles volume friction $\phi$ and motile density $\chi$ fields are chosen in the following manner^37,42^

1 $$V = [\overset{\lower0.5em\hbox{$\smash{\scriptscriptstyle\smile}$}}{u} ({\dot{r}},\dot{z},\dot{t}),\overset{\lower0.5em\hbox{$\smash{\scriptscriptstyle\smile}$}}{w} ({\dot{r}},\dot{z},\dot{t})],\quad T = T({\dot{r}},\dot{z},\dot{t}),\quad C = C({\dot{r}},\dot{z},\dot{t}),\quad N = ({\dot{r}},\dot{z},\dot{t}).$$

The analysis of boundaries exhibition and fore said norms, all the under-conversation equations of energy, momentum, concentration and motile microorganisms are generated for time dependent MHD flow of axisymmetric with Carreau-nanomaterial performances along multiple slip effects and also can be read as.

The Carreau viscosity fluid model resembles with governing flow equations are^37,42–44^.

2 $$ \frac{{\partial \overset{\lower0.5em\hbox{$\smash{\scriptscriptstyle\smile}$}}{u} }}{{\partial {\dot{r}}}} + \frac{{\partial \overset{\lower0.5em\hbox{$\smash{\scriptscriptstyle\smile}$}}{w} }}{{\partial \dot{z}}} + \frac{{\overset{\lower0.5em\hbox{$\smash{\scriptscriptstyle\smile}$}}{u} }}{{{\dot{r}}}} = 0, $$

3 $$ \begin{aligned} & \frac{{\partial \overset{\lower0.5em\hbox{$\smash{\scriptscriptstyle\smile}$}}{u} }}{{\partial \dot{t}}} + \overset{\lower0.5em\hbox{$\smash{\scriptscriptstyle\smile}$}}{u} \frac{{\partial \overset{\lower0.5em\hbox{$\smash{\scriptscriptstyle\smile}$}}{u} }}{{\partial {\dot{r}}}} + w\frac{{\partial \overset{\lower0.5em\hbox{$\smash{\scriptscriptstyle\smile}$}}{u} }}{{\partial \dot{z}}} - \overset{\lower0.5em\hbox{$\smash{\scriptscriptstyle\smile}$}}{v} \frac{{\partial^{2} \overset{\lower0.5em\hbox{$\smash{\scriptscriptstyle\smile}$}}{u} }}{{\partial \dot{z}^{2} }}\left[ {1 + \Gamma^{2} \left( {\frac{{\partial \overset{\lower0.5em\hbox{$\smash{\scriptscriptstyle\smile}$}}{u} }}{{\partial \dot{z}}}} \right)^{2} } \right]^{{\frac{n - 1}{2}}} - \overset{\lower0.5em\hbox{$\smash{\scriptscriptstyle\smile}$}}{v} \left( {n - 1} \right)\Gamma^{2} \frac{{\partial^{2} \overset{\lower0.5em\hbox{$\smash{\scriptscriptstyle\smile}$}}{u} }}{{\partial \dot{z}^{2} }}\left( {\frac{{\partial \overset{\lower0.5em\hbox{$\smash{\scriptscriptstyle\smile}$}}{u} }}{{\partial \dot{z}}}} \right)^{2} \left[ {1 + \Gamma^{2} \left( {\frac{{\partial \overset{\lower0.5em\hbox{$\smash{\scriptscriptstyle\smile}$}}{u} }}{{\partial \dot{z}}}} \right)^{2} } \right]^{{\frac{n - 3}{2}}} + \frac{{\sigma B^{2} \left( t \right)\overset{\lower0.5em\hbox{$\smash{\scriptscriptstyle\smile}$}}{u} }}{\rho } \\ & \quad - \left( {\frac{1}{{\rho_{f} }}} \right)\left[ {\left( {\left( {T - T_{\infty } } \right)\left( {1 - C_{f} } \right)\rho_{f} \beta_{1} g_{1} } \right) - \left( {\left( {\rho_{p} - \rho_{f} } \right)\left( {C - C_{\infty } } \right)g_{1} } \right) - \left( {\left( {\rho_{m} - \rho_{f} } \right)\left( {N - N_{\infty } } \right)g_{1} \gamma_{1} } \right)} \right] = 0, \\ \end{aligned} $$

4 $$ \frac{\partial T}{{\partial \dot{t}}} + u\frac{\partial T}{{\partial {\dot{r}}}} + \overset{\lower0.5em\hbox{$\smash{\scriptscriptstyle\smile}$}}{w} \frac{\partial T}{{\partial \dot{z}}} - \frac{\partial }{{\partial \dot{z}}}\alpha_{m} \left( {\frac{\partial T}{{\partial \dot{z}}}} \right) - \tau \left[ {D_{B} \frac{\partial C}{{\partial \dot{z}}}\frac{\partial T}{{\partial \dot{z}}} + \frac{\partial T}{{\partial \dot{z}}}\frac{{D_{T} }}{{T_{\infty } }}\left( {\frac{\partial T}{{\partial \dot{z}}}} \right)} \right] = 0, $$

5 $$ \frac{\partial C}{{\partial \dot{t}}} + u\frac{\partial C}{{\partial {\dot{r}}}} + \overset{\lower0.5em\hbox{$\smash{\scriptscriptstyle\smile}$}}{w} \frac{\partial C}{{\partial \dot{z}}} - \frac{\partial }{{\partial \dot{z}}}D_{B} \left( {\frac{\partial C}{{\partial \dot{z}}}} \right) - \frac{\partial }{{\partial \dot{z}}}\frac{{D_{T} }}{{T_{\infty } }}\left( {\frac{\partial T}{{\partial \dot{z}}}} \right) = 0, $$

6 $$ \frac{\partial N}{{\partial \dot{t}}} + \overset{\lower0.5em\hbox{$\smash{\scriptscriptstyle\smile}$}}{u} \frac{\partial N}{{\partial {\dot{r}}}} + \overset{\lower0.5em\hbox{$\smash{\scriptscriptstyle\smile}$}}{v} \frac{\partial N}{{\partial \dot{z}}} + \frac{{bW_{c} }}{{C_{w} - C_{\infty } }}\frac{\partial }{{\partial \dot{z}}}\left( {n\frac{\partial C}{{\partial \dot{z}}}} \right) - \frac{\partial }{{\partial \dot{z}}}D_{m} \left( {\frac{\partial N}{{\partial \dot{z}}}} \right) = 0. $$

For all $$(\overset{\lower0.5em\hbox{$\smash{\scriptscriptstyle\smile}$}}{u} ,\,\,\overset{\lower0.5em\hbox{$\smash{\scriptscriptstyle\smile}$}}{w} )$$ denotes the components of velocity for 2-D in $$({\dot{r}},\dot{z})$$ directional profile respectively, $$\Gamma$$ is also constant for the material, $$n$$ is use to represent the power law index, $$v$$ is the viscosity parameter of kinematic energy, $$\rho$$ is the density parameter, The ratio of basic fluid with effect of effective heat capacity of nanoparticles is denoted by $$\tau$$ and $$k,\alpha_{m} ,C_{p} ,D_{T} ,D_{B} ,\dot{t}$$ are the following parameters use in the basic PDE’s in above and denotes the thermal conductivity as well as diffusivity, specific heat, diffusion in thermophoresis as same diffusion in Brownian and last parameter is for time. The boundaries for above PDE’s are written below.

7 $$ \tau = \left( {\frac{{\rho C_{p} }}{{\rho C_{f} }}} \right),\,\,\alpha_{m} = \left( {\frac{k}{{\rho C_{p} }}} \right), $$

8 $$ \left. {\begin{array}{*{20}l} {\overset{\lower0.5em\hbox{$\smash{\scriptscriptstyle\smile}$}}{u} = U_{w} ({\dot{r}},\dot{t}) + \overset{\lower0.5em\hbox{$\smash{\scriptscriptstyle\smile}$}}{u}_{slip} ,\overset{\lower0.5em\hbox{$\smash{\scriptscriptstyle\smile}$}}{w} = 0,} \hfill & {C = C_{w} (x),} \hfill \\ {k\frac{\delta T}{{\delta \dot{z}}} + hf\left( {T_{w} (x) - T} \right) = 0,} \hfill & {N = N_{w} (x),\quad {\text{as}}\quad \dot{z} = 0,} \hfill \\ {\overset{\lower0.5em\hbox{$\smash{\scriptscriptstyle\smile}$}}{u} \to 0,T \to T_{\infty } ,C \to C_{\infty } ,N \to N_{\infty } ,} \hfill & {\dot{z} \to \infty .} \hfill \\ \end{array} } \right\} $$

Then $T_{\infty }$,$C_{\infty }$ and $N_{\infty }$ are the temperature concentration and motile microorganisms at infinity, respectively. With additionally, velocity about partial slip condition is rumored

9 $$ \overset{\lower0.5em\hbox{$\smash{\scriptscriptstyle\smile}$}}{u}_{slip} = l\frac{{\partial \overset{\lower0.5em\hbox{$\smash{\scriptscriptstyle\smile}$}}{u} }}{{\partial \dot{z}}}\left[ {1 + \Gamma^{2} \left( {\frac{{\partial \overset{\lower0.5em\hbox{$\smash{\scriptscriptstyle\smile}$}}{u} }}{{\partial \dot{z}}}} \right)^{2} } \right]^{{\frac{n - 1}{2}}} . $$

In the above equation $l$ is the slip dimension for length. The non-dimensional appropriate parameters are

10 $$ \zeta = \frac{{\dot{z}}}{{{\dot{r}}}}{\text{R}}_{e}^{\frac{1}{2}} ,\quad \psi \left( {{\dot{r}},\dot{z},\dot{t}} \right) = - {\dot{r}}^{2} U_{w} {\text{R}}_{e}^{{ - \frac{1}{2}}} f(\zeta ), $$

11 $$ \theta \left( \zeta \right) = \frac{{T - T_{\infty } }}{{T_{w} (x) - T_{\infty } }},\quad \phi \left( \zeta \right) = \frac{{C - C_{\infty } }}{{C_{w} (x) - C_{\infty } }},\quad \chi \left( \zeta \right) = \frac{{N - N_{\infty } }}{{N_{w} (x) - N_{\infty } }}. $$

For all $$\psi$$ is the stream function advent by ‘Stokes’ with the stuff $$(\overset{\lower0.5em\hbox{$\smash{\scriptscriptstyle\smile}$}}{u} ,\overset{\lower0.5em\hbox{$\smash{\scriptscriptstyle\smile}$}}{w} ) = \left( { - \frac{1}{{{\dot{r}}}}\frac{\partial \psi }{{\partial \dot{z}}},\frac{1}{{{\dot{r}}}}\frac{\partial \psi }{{\partial {\dot{r}}}}} \right),\;(\theta ,\phi ,\chi )$$ the dimensionless thickness of boundary layers, volume friction of nanoparticle and motile microorganisms respectively. The components of velocity are

12 $$ \overset{\lower0.5em\hbox{$\smash{\scriptscriptstyle\smile}$}}{u} = U_{w} f^{\prime}\left( \zeta \right),\overset{\lower0.5em\hbox{$\smash{\scriptscriptstyle\smile}$}}{w} = - 2U_{w} {\text{R}}_{e}^{{ - \frac{1}{2}}} f\left( \zeta \right). $$

Relieving Eq. (11) in to Eqs. (3), (4), (5) and (6), developed the following non-linear ODE’s

13 $$ \begin{aligned} & \left\{ {1 + W_{e}^{2} \left( {f^{\prime\prime}} \right)^{2} } \right\}^{{\frac{n - 2}{2}}} \left\{ {1 + nW_{e}^{2} \left( {f^{\prime\prime}} \right)^{2} } \right\}(f^{\prime\prime\prime}) + 2ff^{\prime\prime} - \left( {f^{\prime}} \right)^{2} - M^{2} f^{\prime} \\ & \quad + \lambda (\theta - Nr\phi - Nc\chi ) - \left[ {f^{\prime} + \frac{\zeta }{2}f^{\prime\prime}} \right]K^{*} = 0, \\ \end{aligned} $$

14 $$ \theta^{\prime\prime} + \left\{ {2f\theta ^{\prime} - \frac{{K^{ * } }}{2}\zeta \theta^{\prime} + N_{b} \theta^{\prime}\phi^{\prime} + N_{t} \left( {\theta^{\prime}} \right)^{2} } \right\}{\text{P}}_{r} = 0, $$

15 $$ \phi^{\prime\prime} + 2S_{c} f\phi^{\prime} - \frac{{K^{ * } }}{2}S_{c} \zeta \phi^{\prime} + \frac{{N_{t} }}{{N_{b} }}\theta^{\prime\prime} = 0, $$

16 $$ \chi ^{\prime\prime} + {\text{P}}_{r} L_{b} f\chi^{\prime} - P_{e} \left( {\phi^{\prime}\chi^{\prime} + \phi^{\prime\prime}\left( {\chi + \delta } \right)} \right) = 0, $$

17 $$ \left. \begin{gathered} f^{\prime}\left( 0 \right) = 1 + \beta f^{\prime\prime}\left( 0 \right)\left\{ {1 + W_{e}^{2} \left( {f^{\prime\prime}\left( 0 \right)} \right)^{2} } \right\}^{{\frac{n - 1}{2}}} ,\;f\left( 0 \right) = 0,\;\theta ^{\prime}\left( 0 \right) = - \gamma \left( {1 - \theta \left( 0 \right)} \right),\;\phi \left( 0 \right) = 1,\;\chi (0) = 1, \hfill \\ f{\prime }\left( \infty \right) \to 0,\theta \left( \infty \right) \to 0,\;\phi \left( \infty \right) \to 0,\;\chi \left( \infty \right) \to 0. \hfill \\ \end{gathered} \right\} $$

The above Eqs. (13–16) are having the following dimension-less parameters namely Biot number, local Weissenberg number^20^, unsteadiness parameter, Schmidt number, thermophoresis parameter, Magnetic parameter, generalized Biot number, velocity slip parameter, Lewis number, Bio-convection rely number, Bouncy ratio parameter, Bio convective Lewis number, Peclet number, Brownian motion parameter, Prandtl number respectively are given below

18 $$ \left. \begin{gathered} Bi = \frac{{h_{f} }}{{k_{1} }}\sqrt {\frac{\nu }{{ \wedge_{1} }}} ,\;W_{e}^{2} = \frac{{\Gamma^{2} \wedge_{1}^{3} {\dot{r}}^{2} }}{{v\left( {1 - \wedge_{2} \dot{t}} \right)^{3} }},\;A = \frac{{ \wedge_{2} }}{{ \wedge_{1} }},\;{\text{P}}_{r} = \frac{v}{{\alpha_{m} }},\;S_{c} = \frac{v}{{D_{B} }},\;N_{t} = \frac{{\tau D_{T} \left( {T_{w} - T_{\infty } } \right)}}{{vT_{\infty } }},\;N_{b} = \frac{{\tau D_{B} \left( {C_{w} - C_{\infty } } \right)}}{v}, \hfill \\ M^{2} = \frac{{\sigma D_{0}^{2} }}{{\rho \wedge_{1} }},\;\gamma = \frac{{{\dot{r}}h_{f} }}{K}{\text{R}}_{e}^{{ - \frac{1}{2}}} ,\;\beta = \frac{l}{{{\dot{r}}}}{\text{R}}_{e}^{\frac{1}{2}} ,\;\delta = \frac{{N_{\infty } }}{{N_{w} (x) - N_{\infty } }},\;N_{c} = \frac{{\gamma (\rho_{m} - \rho_{f} )(n_{w} - n_{\infty } )}}{{\beta \rho_{f} (1 - C_{\infty } )T_{\infty } }}, \hfill \\ N_{r} = \frac{{(\rho_{p} - \rho_{f} )(C_{w} - C_{\infty } )}}{{\beta \rho_{f} (1 - C_{\infty } )T_{\infty } }},\;L_{b} = \frac{\nu }{{D_{nn} }},\;P_{e} = \frac{{bW_{c} }}{{D_{mn} }}, \hfill \\ \end{gathered} \right\} $$

The system of mass, flow and heat transfer are the local skin friction coefficient $C_{f}$ , the local Nusselt number $N\mu_{r}$, local Sherwood number $Sh_{r}$ and the motile microorganism’s number which are written as

19 $$ C_{f} = \frac{{\tau_{w} |_{{\dot{z} = 0}} }}{{\rho U_{w}^{2} }},\;Nu_{r} = \frac{{{\dot{r}}q_{w} |_{{\dot{z} = 0}} }}{{K\left( {T_{w} - T_{\infty } } \right)}},\;Sh_{r} = \frac{{rq_{m} |_{{\dot{z} = 0}} }}{{D_{B} \left( {C_{w} - C_{\infty } } \right)}},\;Nn_{r} = \frac{{xq_{n} |_{{\dot{z} = 0}} }}{{D_{N} (N_{W} - N_{\infty } )}}. $$

The wall ‘shear stress’ as same as ‘heat flux’ and ‘mass flux’ respectively are in Eq. (20)

20 $$ \tau_{w} = \mu_{0} \frac{{\partial \overset{\lower0.5em\hbox{$\smash{\scriptscriptstyle\smile}$}}{u} }}{{\partial \dot{z}}}\left[ {1 + \Gamma^{2} \left( {\frac{{\partial \overset{\lower0.5em\hbox{$\smash{\scriptscriptstyle\smile}$}}{u} }}{{\partial \dot{z}}}} \right)^{2} } \right]_{{\dot{z} = 0}}^{{\frac{n - 1}{2}}} ,\;q_{w} = - k\left( {\frac{\partial T}{{\partial \dot{z}}}} \right)_{{\dot{z} = 0}} ,\;q_{n} = - D_{B} \left( {\frac{\partial C}{{\partial \dot{z}}}} \right)_{{\dot{z} = 0}} . $$

The solution of Eqs. (9), (18) and (19), the slog, heat and mass transfer rates get the following form:

21 $$ {\text{R}}_{e}^{\frac{1}{2}} Cf = f^{\prime \prime }(0)\left[ {1 + W_{e}^{2} \left( {f^{\prime \prime }(0)} \right)^{2} } \right]^{{\frac{n - 1}{2}}} ,\;{\text{R}}_{e}^{{ - \frac{1}{2}}} Nu = - \theta (0),\;{\text{R}}_{e}^{{ - \frac{1}{2}}} Sh = - \phi^ {\prime} (0). $$

Then the local Reynolds number is ${\text{R}}_{e} = \left( {\frac{{{\dot{r}}U_{w} }}{v}} \right).$

## Numerical technique

**Figure 2:**
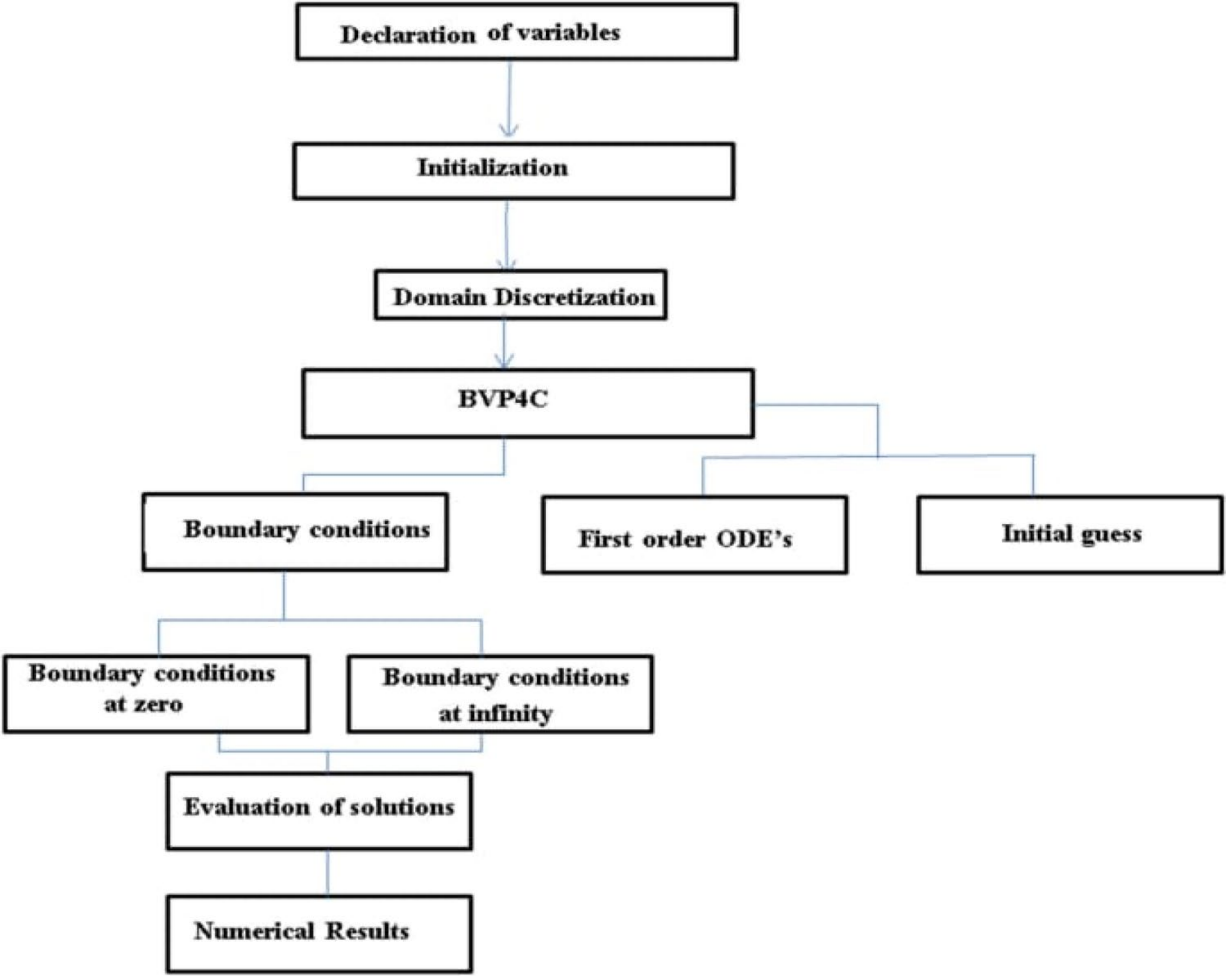
Flow chart of numerical scheme (Bvp4c).

Physically, it is hard to find the exact solution of the system of effective non-linear ODE’s (13)–(14) with the boundaries (15) and (16). Thus, nonlinear ODE’s having equations like, energy, momentum, nanoparticles volume friction and motile microorganisms along with the suitable boundaries are numerically solved by using the bvp4c shooting technique. In this technique boundary-value problems are turned into an initial-value problem. As such, it is possible to perform the following procedure: first, in order to determine an initial value of a parameter, one should use the bvp4c method; second, to adjust the initial value of the parameter so that the solution satisfies the boundary conditions. In the following form of initial value problems, this technique is considered to be suitable as shown in Fig. 2.

22 $$\frac{dy}{{dx}} = f\left( {x,y} \right),g\left( {x_{i} } \right) = g_{i} .$$

For numerical purpose the differential Eqs. (13)–(16) are first transformed into a system of seven first order differential equations. To run the code by using bvp4c, seven original assumptions but four initial assumptions each in $f^{\prime}(\infty ) = 0,\,\theta (\infty ) = 0,\,\,\,\phi (\infty ) = 0\,\,\,$ and $\,\chi (\infty ) = 0$ are nonentities. These four end assumptions are used to advance four unidentified original assumptions with effect of shooting technique. Newton–Raphson Method is very useful for finding the initial guess. Thus we have

23 $$g^{\prime}_{1} = g_{2,} g_{2} = g_{3} ,g^{\prime}_{3} = \frac{{g_{2}^{2} - 2g_{1} g_{2} + A\left( {g_{2} + \frac{\zeta }{2}g_{3} } \right) + M^{2} g_{2} }}{{\left\{ {1 + nW_{e}^{2} g_{3}^{2} } \right\}\left\{ {1 + W_{e}^{2} g_{3}^{2} } \right\}}},$$

24 $$g^{\prime}_{4} = g_{5} ,\,g^{\prime}_{5} = {\text{P}}_{r} \left\{ { - 2g_{1} g_{5} + \frac{A}{2}\zeta g_{5} - N_{b} g_{5} g_{7} - N_{t} \left( {g_{5} } \right)^{2} } \right\},$$

25 $$g^{\prime}_{6} = g_{7} ,g^{\prime}_{7} = - 2S_{c} g_{1} g_{7} + \frac{A}{2}S_{c} \zeta g_{7} - \frac{{N_{t} }}{{N_{b} }}g^{\prime\prime}_{5} ,$$

26 $$g^{\prime}_{9} = P_{e} \left[ {g_{8}^{\prime } \left( {g_{9} + \delta_{1} } \right) + g_{7} g_{9} } \right] - P_{r} L_{b} g_{1} g_{9} ,$$

where the unknowns are stated as

27 $$f = g_{1} ,f^{\prime} = g_{2} ,f^{\prime\prime} = g_{3} ,\theta = g_{4} ,\theta^{\prime} = g_{5} ,\phi = g_{6} ,\phi^{\prime} = g_{7} ,\,\,\chi = g_{8} ,\chi^{\prime} = g_{9} .$$

The boundary conditions are

28 $$\left. \begin{gathered} g_{1} (0) = 0,\;0 = g_{2} (0) - 1 - \beta g_{3} (0)\left\{ {1 + W_{e}^{2} (g_{3} (0))^{2} } \right\}^{{\frac{n - 1}{2}}} ,\;g_{5} (0) = - \gamma (1 - g_{4} (0)),\;g_{6} (0) = 1, \hfill \\ g_{2} (\infty ) \to 0,\;g_{4} (\infty ) \to 0,\;g_{6} (\infty ) \to 0. \hfill \\ \end{gathered} \right\}$$

## Numerical analysis

The evaluations between the current and previously available numerical results makes effective contract in Tables 1 and 2 the computational and numerical results at the same values reflected the same nature at magnetic $M$ and velocity slip factors $\beta$. A numerical computation for time dependent MHD axisymmetric flow of Carreau nanomaterial performances over motile microorganisms is performed. This numerical work is achieved by chasing the active numerical pattern which known as the shooting technique along bvp4c method with help of computational software Matlab (bvp4c). This study disclose the behavior of several nion-dimensional parameters like power law index n, magnetic factor $M$, local Weissenberg number $W_{e}$, velocity slip factor $\beta$, generalized Biot number $B_{i}$, unsteadiness parameter A, Rayleigh number $N_{c}$, buoyancy parameter $N_{r}$, Prandtl number ${\text{P}}_{r}$ and Schmidt number $S_{c}$. The numerical computation of local skin friction coefficient, local Nusselt number, local Sherwood number and also motility number in Tables 3, 4, 5 and 6 respectively elaborates the major and necessary variation of power law index on the two different values in the comparison of the different factors depict a slightly growth of the results same as the factor effects of each variation with the rises of power law index 0.5–1.5 and also makes declaim effect in both case of power law index of 0.5 and 1.5 of the Lewis number $\delta$, Weissenberg number $W_{e}$, Bio-convective Lewis number $L_{b}$, Biot number $\gamma$ and buoyancy parameter $N_{r}$ with effective growth in the values of respective factors but in the other hand magnetic factor $M$ and Rayleigh number $N_{c}$ shows invers effect of the other under discussion factors but Lewis number $\delta$, Biot number $\gamma$ and buoyancy parameter $N_{r}$ with effective growth in the values of respective factors but in the other hand Prandtl factor $P_{r}$ and Rayleigh number $N_{c}$ shows invers effect of the other under discussion factors then only the Rayleigh number $N_{c}$ but in the other hand the other under discussion factors Lewis number $\delta$, Peclet number $P_{e}$ and buoyancy parameter $N_{r}$ shows the effective growth in the results of respective factors at the end Rayleigh number $N_{c}$ and Prandtl factor $P_{r}$ with effective growth in the values of respective factors but in the other hand Bioconvective Lewis number $L_{b}$, Biot number $\gamma$ and buoyancy parameter $N_{r}$ shows an incremental effect of these respective factors in the respectively tables.

**Table 1:** Assessment of numerical computational results $- f^{\prime\prime}(0)$ for different values of the magnetic factor $M^{2}$.

$M^{\,\,\,\,\,\,\,\,2}$	Makinde et al.^35^	Azam et al.^37^	Present results
0	1.17372	1.17372	1.17372
0.5	1.36581	1.36581	1.36581
1	1.53571	1.53571	1.53571
2	1.83049	1.83049	1.83049
3	2.08484	2.08485	2.08485

**Table 2:** Numerical and computational results for accuracy check of $- f^{\prime\prime}(0)$ for different values of the velocity slip factor β.

$\beta$	$- f^{\prime\prime}(0)$					
	Exact^38^	HPM^38^	Perturbation^38^	Asymptotic^38^	RK-45^37^	Present (Bvp4c)
0	1.173721	1.178511	1.173721		1.173734	1.173734
0.1	1.153472	1.157311	1.153489		1.153485	1.153485
0.2	1.134017	1.136998	1.13409		1.134031	1.134031
0.5	1.079949	1.08082	1.08101		1.079964	1.079964
0.1	1.001834	1.000308	1.009522		1.001850	1.001850
0.2	0.878425	0.874453	0.930213		0.878444	0.878444
0.5	0.650528	0.645304	1.201623	1.529918	0.650550	0.650550
1	0.46251	0.458333		0.574163	0.462547	0.462547
2	0.29905	0.296532		0.310715	0.299099	0.299099
5	0.149393	0.148454		0.14959	0.149455	0.149455
10	0.082912	0.082532		0.082833	0.082974	0.082974
20	0.044368	0.044228		0.044337	0.044423	0.044423
50	0.018732	0.018698		0.018727	0.018770	0.018770

**Table 3:** Numerical analysis of skin friction coefficient $- f^{\prime\prime}(0)$ for the values of δ.

$\delta$	$M$	$W_{e}$	$\lambda$	$\gamma$	$N_{r}$	$N_{c}$	$- f^{\prime\prime}(0)$	
0.1	0.1	3	0.1	1	0.5	0.2	n = 0.5	n = 1.5
0.2							0.5094	0.5103
0.3							0.5093	0.5102
0.4							0.5092	0.5101
	0.3						0.5261	0.5271
	0.5						0.5403	0.5501
	0.7						0.5543	0.5581
		2					0.5094	0.5103
		4					0.5093	0.5102
		6					0.5092	0.5101
			0.2				0.4972	0.4982
			0.3				0.4857	0.4865
			0.4				0.4749	0.4761
				2			0.3165	0.3175
				3			0.2312	0.2325
				4			0.1826	0.1842
					0.4		0.5195	0.5198
					0.8		0.5095	0.5109
					1		0.5099	0.5100
						0.4	0.5072	0.5100
						0.8	0.5164	0.5174
						1	0.5313	0.5323

**Table 4:** Numerical analysis of Local Nusselt number $\theta^{\prime}(0)$ for the values of δ.

$\delta$	$\lambda$	$N_{r}$	$N_{c}$	$\theta^{\prime}(0)$	
0.1	0.1	0.5	0.5	n = 0.5	n = 1.5
0.2				0.5094	0.5103
0.3				0.5093	0.5102
0.4				0.5092	0.5101
	0.2			0.2438	0.2451
	0.3			0.2531	0.2583
	0.4			0.2609	0.2624
		0.4		0.2335	0.2425
		0.8		0.2266	0.2282
		1		0.2212	0.2231
			0.4	0.2343	0.2352
			0.8	0.2244	0.2362
			1	0.2182	0.2367

**Table 5:** Numerical analysis of Sherwood number $\phi^{\prime}(0)$ for the values of $L_{e}$.

$L_{e}$	$N_{r}$	$N_{c}$	$\phi^{\prime}(0)$	
2	0.5	0.5	n = 0.5	n = 1.5
1			0.5611	0.6112
3			0.5806	0.6114
5			0.5788	0.6116
	0.4		0.5835	0.5880
	0.8		0.5665	0.5689
	1		0.5529	0.5540
		0.4	0.5858	0.5890
		0.8	0.5610	0.5640
		1	0.5455	0.5465

**Table 6:** Numerical analysis of motile microorganisms $\chi^{\prime}(0)$ for the values of $L_{b}$.

$L_{b}$	${\text{P}}_{r}$	$N_{r}$	$N_{c}$	$\chi^{\prime}(0)$	
2	1.2	0.5	0.5	n = 0.5	n = 1.5
2				0.4312	0.4156
3				0.4218	0.3919
4				0.4144	0.3846
	1			0.4127	0.3455
	3			0.4043	0.3356
	5			0.3964	0.3141
		0.4		0.4112	0.3956
		0.8		0.4204	0.387
		1		0.4292	0.3835
			0.4	0.4146	0.3987
			0.8	0.4070	0.3874
			1	0.4024	0.3821

Tables 1 and 2 show the comparison of existing study with already published results and there seems a good correlation among them. It is observed that buoyancy parameter $N_{r}$ is presented a decreases in the results with increment in the values with $- f^{\prime\prime}(0)$ and $\theta^{\prime}(0)$ but Rayleigh number $N_{c}$ presented inverse behavior in both discussed cases. On the other hand buoyancy parameter $N_{r}$ is presented an increase in the results with increment in the values with $\phi^{\prime}(0)$ and $\chi^{\prime}(0)$ but Rayleigh number $N_{c}$ presented inverse behavior in both discussed cases.

## Numerical section

See Tables 1−6.

## Graphical analysis

**Figure 3:**
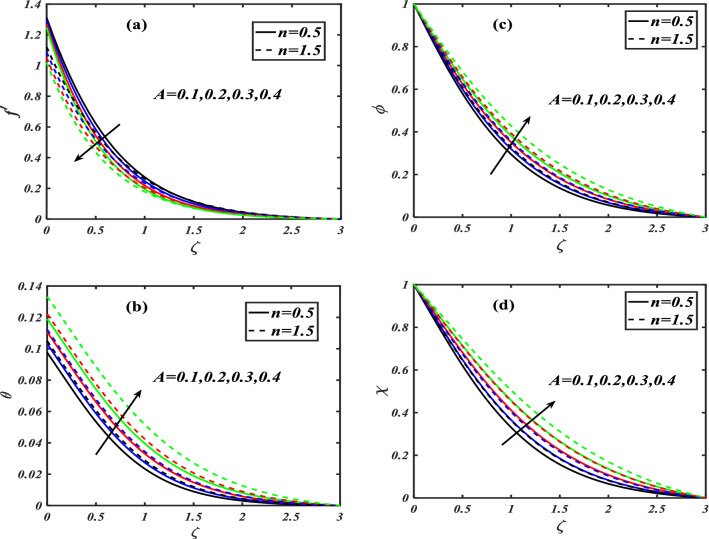
(**a**–**d**) Illustrating the effect of unsteadiness parameter on $f'(\zeta ),\theta (\zeta ),\phi (\zeta )\& \chi (\zeta )$.

**Figure 4:**
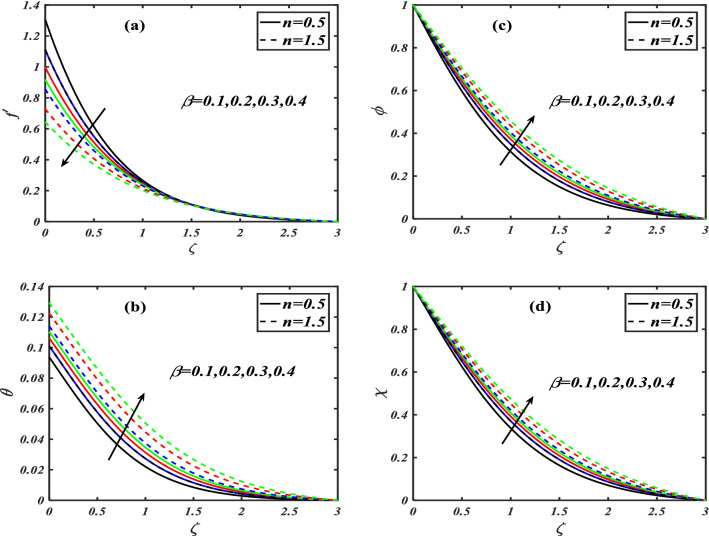
(**a**–**d**) Illustrates the effect of $\beta$ on $f'(\zeta ),\theta (\zeta ),\phi (\zeta )\& \chi (\zeta )$.

**Figure 5:**
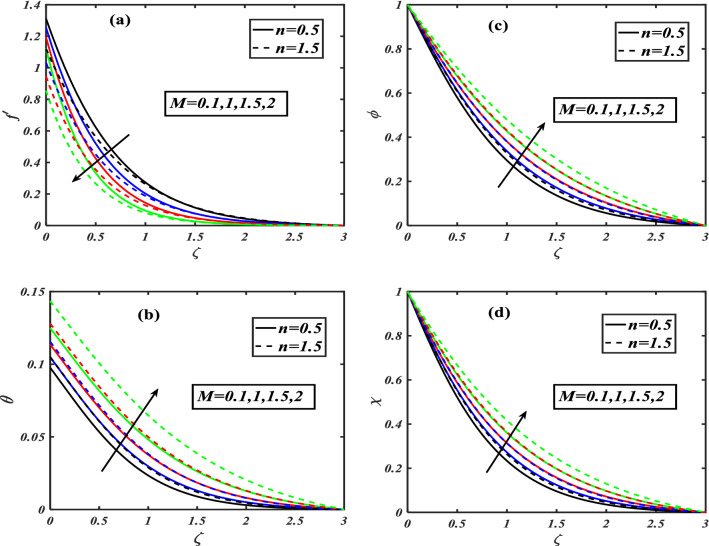
(**a**–**d**) Illustrates the effect of magnetic parameter on $f'(\zeta ),\theta (\zeta ),\phi (\zeta )\& \chi (\zeta )$.

**Figure 6:**
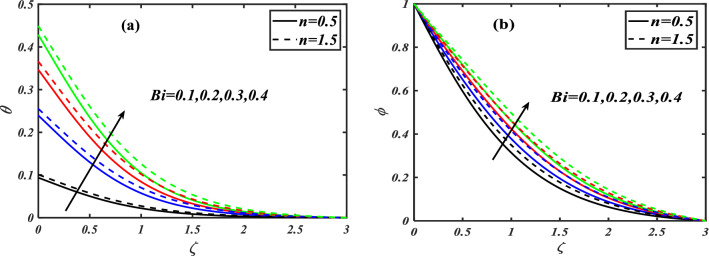
(**a**, **b**) Illustrates the effect of Biot number on $\theta (\zeta ),\,\,\phi (\zeta )$.

**Figure 7:**
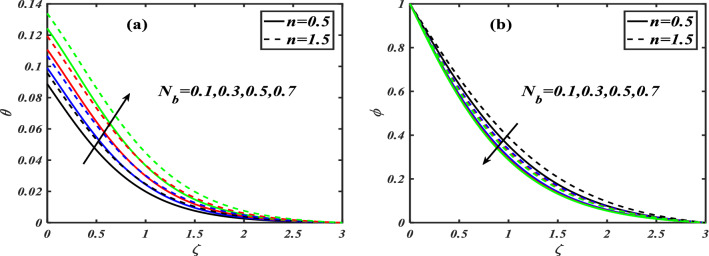
(**a**, **b**) Illustrating the impact of Brownian motion parameter over $\theta (\zeta ),\phi (\zeta )$.

**Figure 8:**
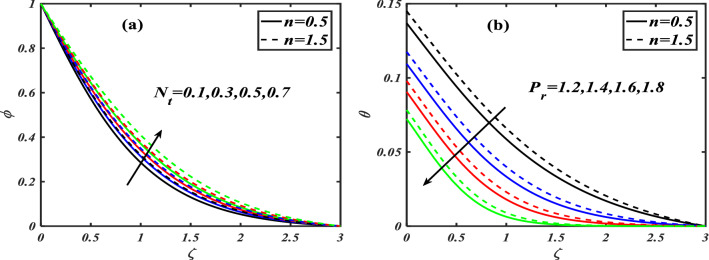
(**a**) Depicts thermophoresis on $\phi (\zeta )$ while (**b**) depicts Prandtl number on $\theta (\zeta )$.

**Figure 9:**
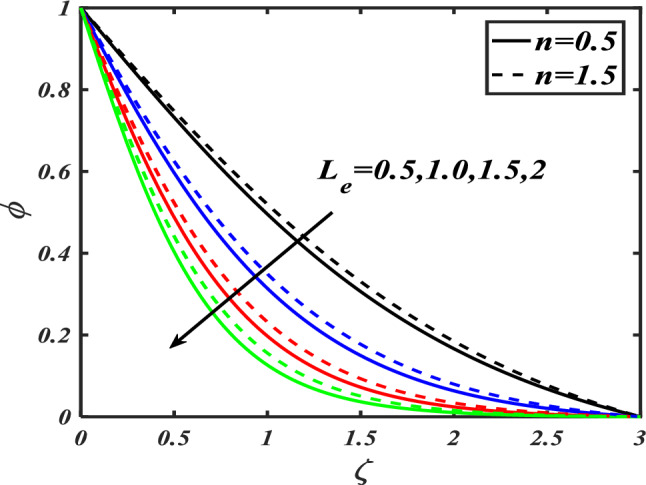
Depicts the effect of Lewis number on $\phi (\zeta )$.

**Figure 10:**
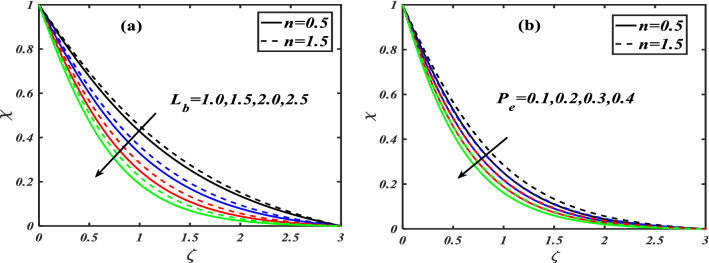
(**a**) Depicts the effect of $L_{b}$ while (**b**) depicts $P_{e}$ on $\chi (\zeta )$.

Here we will discuss the different behaviors of axisymmetric flow with Carreau-nano fluid and motile microorganisms with the multi slip effect on time dependent MHD flow. Here we will check the different parameters effects on $f^{\prime}(\zeta ),\,\,\theta (\zeta ),\,\,\phi (\zeta )\,\,{\text{and}}\,\,\,\chi (\zeta )$, of non-Newtonian fluids. The Fig. 3a–d depict the behavior of unsteadiness parameter $A$ on temperature, velocity, concentration and motile density distribution on non-Newtonian fluids e.g. dilatant and pseudo plastic fluids $\,n = 0.5$ and $\,n = 1.5$, respectively. Figure 3a is sketched to investigate the behavior of unsteadiness parameter on velocity field. It is observed that velocity distribution shows decrement when the unsteadiness parameter is uplifted for both the fluids i.e. Dilatant and pseudo plastic fluids $\,n = (0.5,1.5)$. Actually, here $\,n = (0.5,1.5)$ are the non-Newtonian fluids, which means their shear stress and shear rate are not equally distributed. This makes it more viscous and it causes cutback in velocity. Viscosity is inversely proportion to velocity that’s decreases its velocity. Figure 3b is depicting the behavior on temperature. It is revealed that the temperature coefficient gets arise when the values of unsteadiness parameter is gradually increases on both of the fluid, $\,n = 0.5$ and $\,n = 1.5$, Dilatant and Pseudo plastic fluids. Physically, it is noticed that the non-Newtonian fluids have more viscosity coefficient that causes increment in temperature field. Figure 3c illustrates that how concentration coefficient is affected by unsteady parameter A. It is observed that with the increase in unsteady parameter it results a rise in concentration field. The main reason for behaving like this is due to viscosity because viscosity parameter increases concentration distribution of nanoparticle fluids. For testing the behavior of motile density on unsteadiness parameter Fig. 3d is sketched. It is observed that when we increase the value of unsteady parameter in shear thinning and shear thickening fluid the motile density parameter uplifted. It can assume that bio molecules that are in the base fluid become more dense then heat dissipation and transfer become high. The Fig. 4a–d depict the behavior of velocity slip parameter $\beta$ that increases in the slip condition makes the positive effect in the geometrically and graphically in this group of graphs the variation of $f^{\prime}(\zeta )$ which makes the down lift the curve and in others $\theta (\zeta ),\phi (\zeta )$ and $\chi (\zeta )$ respectively shows up lift behavior. Velocity slip parameter has vital role in heat transfer rate as it cause an uplift in all the parameters except velocity it is due to the increase of mass transfer rate which make the base fluid more dense and it carry less heat so its velocity field become low, in the same phenomenon concentration distribution and motility of the base fluid increases due to same factor. Figure 5a–d are depicting the behavior of magnetic parameter on velocity, boundary layer thickness, volume friction and motile density distribution in Dilatant and Pseudo plastic fluids $\,n = 0.5$ and $\,n = 1.5$. Figure 5a illustrates the behavior of magnetic parameter on velocity. It is inspected that velocity parameter show reduction as the value of magnetic effect increases. The reason behind this decrement is basically the fluid which is magnetic, since the magnetic effect create an impact on the nanoparticles inside the fluid, and net forces become heavy which causes a decrease in fluid velocity while testing temperature field Fig. 5b is drawn for the evidence. It is observed that temperature of the nanoparticle fluid increases as the value of magnetic effect increases. The main reason for uplifting of the temperature is, magnetic parameter directly effects on the nanoparticle fluid either it is shear thinning or shear thickening. Figure 5c, d concentration profile and motile density distribution have same characteristic as temperature do. It is observed that with the increment in magnetic effect it gives a rise in concentration and motile density distribution for both dilatant and pseudo plastic fluids. Figure 6a, b illustrates the effect of Biot number Bi on concentration profile. It is noted that Biot number directly effect on concentration profile. The rise in Biot number gives a clear rise in concentration in both dilatant and pseudo plastic fluids. Figure 7a, b depicts the effects of Brownian motion $Nb$ on temperature and concentration profile. It is seen that when the value of Brownian motion is increased it gives a rise in temperature as Brownian motion directly effects on temperature for both $\,n = 0.5$ and $\,n = 1.5$. While in case of testing concentration profile in behaves opposite to temperature, it is seen that concentration profile falls down when the value of brown motion is increased for both the cases $\,n = 0.5$ and $\,n = 1.5$. Thermophoresis is the phenomenon which is the cause that makes nanoparticles of the fluid flow from hot region to cold region. In testing with dilatant and pseudo plastic fluids the behavior of thermophoresis on volume friction is inspected with Fig. 8a. It exhibits that in shear thickening and shear thinning fluids concentration field increases with the incrimination of thermophoresis. Prandtl number shows the fluctuation only on temperature distribution shown in Fig. 8b. In Fig. 9, Lewis number decreases the temperature of nanoparticle dilatant and pseudo fluids when it is uplifted. Lewis number also has very effective behavior while reacting in dilatant and pseudo plastic fluids. It is noticed that Lewis number decreases the effect of volume friction as it grows up illustrating Fig. 10a as evidence. For the inspection of bio-convection Lewis number on motile density distribution Fig. 10b is drawn. It is clearly visualized that Peclet number devaluates the motile density as we elevate Peclet number.

## Graphical section

See Figs. 3–10.

## Conclusion

- Impact of multiple slip on magnetized time dependent axisymmetric flow on Carreau nanofluid with motile microorganisms is observed in this study. The major findings of the current investigation is given below:
- The enhancement in the values of unsteady parameter *A*, velocity slip parameter *β*, magnetic parameter *M* the velocity profile decline.
- The temperature profile boosted up on increment in the values of the Biot number $B_{i}$, Thermophoresis parameter $N_{t}$, magnetic parameter $M$ and slip velocity Parameter *A*.
- As magnifying intensity in the values of the unsteady parameter *A*, velocity slip parameter $\beta$ and magnetic parameter *M*.
- The nanoparticles concentration profile increases but decreasing as enhance the values of the Brownian motion parameters $N_{b}$.
- The density of the motile microorganism’s retard as intensity in the values of the Peclet number $P_{e}$ and bio-convection Lewis number $L_{b}$.

## Data Availability

The datasets used and/or analysed during the current study available from the corresponding author on reasonable request.
